# Hepatitis B virus infection in Nigeria: a systematic review and meta-analysis of data published between 2010 and 2019

**DOI:** 10.1186/s12879-021-06800-6

**Published:** 2021-10-30

**Authors:** Busayo I. Ajuwon, Isabelle Yujuico, Katrina Roper, Alice Richardson, Meru Sheel, Brett A. Lidbury

**Affiliations:** 1 Research School of Population Health, ANU College of Health and Medicine, The Australian National University, Acton, ACT Australia; 2 Department of Biosciences and Biotechnology, Faculty of Pure and Applied Sciences, Kwara State University, Malete, Nigeria; 3 Statistical Consulting Unit, The Australian National University, Acton, ACT Australia; 4 Department of Health Evidence, TRadboud UMC, 6500 HB Nijmegen, The Netherlands

## Abstract

**Background:**

Hepatitis B virus (HBV) is an infectious disease of global significance, causing a significant health burden in Africa due to complications associated with infection, such as cirrhosis and liver cancer. In Nigeria, which is considered a high prevalence country, estimates of HBV cases are inconsistent, and therefore additional clarity is required to manage HBV-associated public health challenges.

**Methods:**

A systematic review of the literature (via PubMed, Advanced Google Scholar, African Index Medicus) was conducted to retrieve primary studies published between 1 January 2010 and 31 December 2019, with a random-effects model based on proportions used to estimate the population-based prevalence of HBV in the Nigerian population.

**Results:**

The final analyses included 47 studies with 21,702 participants that revealed a pooled prevalence of 9.5%. A prevalence estimate above 8% in a population is classified as high. Sub-group analyses revealed the highest HBV prevalence in rural settings (10.7%). The North West region had the highest prevalence (12.1%) among Nigeria’s six geopolitical zones/regions. The estimate of total variation between studies indicated substantial heterogeneity. These variations could be explained by setting and geographical region. The statistical test for Egger’s regression showed no evidence of publication bias (*p* = 0.879).

**Conclusions:**

We present an up-to-date review on the prevalence of HBV in Nigeria, which will provide critical data to optimise and assess the impact of current prevention and control strategies, including disease surveillance and diagnoses, vaccination policies and management for those infected.

**Supplementary Information:**

The online version contains supplementary material available at 10.1186/s12879-021-06800-6.

## Background

Hepatitis B virus (HBV) is an *Hepadnavirus* that infects liver cells, and was responsible for an estimated 820,000 deaths in 2019; for example, from the subsequent development of hepatocellular carcinomas (HCC) [1]. An estimated 3.6% of the global population is affected by chronic HBV infection [2, 3]. Although viral hepatitis is a significant public health problem globally, it has not been prioritised until recently. In 2016, the World Health Organisation (WHO) adopted the Global health sector strategy on viral hepatitis, which set the goal of eliminating viral hepatitis as a public health problem by 2030, and specifically for 90% of infected persons to be diagnosed by 2030 [4]. Similarly, the 67th World Health Assembly of the WHO on viral hepatitis prevention and control recently reaffirmed the importance of monitoring viral hepatitis prevention, diagnosis, and treatment progress both nationally and globally [5].

In Africa, approximately 60 million people live with chronic HBV infection with an estimated prevalence of 6.2% [6]. New infection rates are highest among children, and transmission predominantly occurs via perinatal routes. The global prevalence of chronic HBV infection among children under five years declined from 5% in the pre-vaccine era (1980s to early 2000s) to less than 1% in 2019. [1, 7]. Vaccination to protect against HBV infection is part of the WHO Extended Programme for Immunisation (EPI) and has been progressively rolled out across Africa since 1995, alongside enhanced interventions for the prevention of mother to child transmission. Despite more than two decades of vaccine introduction which has been critical for reducing infections in children, the overall population prevalence of HBV infection remains high across many settings in SSA (> 8%) [1, 8, 9]. Early epidemiological studies have suggested a high variation in the estimates of HBV prevalence between countries and subgroups of the population in SSA. These variations are often explained by methodological differences [10, 11].

In Africa, Nigeria is ranked as one of the countries that is hyper-endemic for HBV infection (> 8%) [12]. Approximately nine in ten Nigerians who live with chronic HBV are unaware of their infection status, and are missing from the global public health statistics due to a lack of resources, awareness, and political will for addressing Nigeria’s HBV plight [13–15]. Consequently, Nigeria has one of the highest rates of HBV-attributable cancer in West Africa, with an age-standardised incidence estimate of 2.6 to < 5.1 cases per 100,000 person-years [16, 17]. HCC is a highly aggressive cancer with limited treatment options, often lacking in resource-constrained settings [18]. The lack of affordable diagnostics—for example specialised immunoassays and nucleic acid tests, as well as the out-of-pocket cost for vulnerable populations, constitute potential barriers to eliminating viral hepatitis B in Nigeria, thus making HBV a significant threat to public health. Further, clinical and epidemiological research on HBV infection in Nigeria are developing, but have not been able to attract appropriate funding and investment.

There are no up-to-date country-wide systematic reviews reporting HBV prevalence in Nigeria. The first systematic review and meta-analysis of HBV in Nigeria was conducted in 2013 [19] and included 61% of articles published before 2010. Further, the systematic review conducted by Schweitzer et al. [8] on the estimations of the worldwide prevalence of chronic HBV infection was limited in scope, since the study did not provide detailed information on the sources of data for each country; the review only estimated the prevalence without emphasis on at-risk sub-groups and specific populations to which interventions should be mostly directed. These limitations suggest a research gap that requires an up-to-date comprehensive investigation. To address this, we conducted a systematic review to estimate the prevalence of HBV in the Nigerian population. Updated national and sub-national data are also needed to design targeted control and prevention strategies to HBV infections.

## Methods

### Study setting

Nigeria is a densely populated country in Africa, with a population density of 226 people per km^2^. It has the largest population in Africa, with approximately 206 million people in an area of 923,679 km^2^, and is located in West Africa on the Gulf of Guinea, between Benin and Cameroon [20, 21]. Nigeria is a federation of 36 states and the Federal Capital Territory, grouped into six geopolitical zones. Nigeria’s economy is the largest in Africa, with nominal GDP and purchasing power parity of $450 billion and $1 trillion, respectively. However, its Human Development Index ranks 158th globally, and the country is classified as a lower-middle-income economy, with a gross national income per capita between $1036 and $4045 [22]. The total healthcare expenditure was estimated at $18.3 billion in 2014, and the primary source has been out-of-pocket expenditure, constituting about 70.3% of the total healthcare expenditure in 2009 [23]. The expenditure of the Government as a percentage of GDP is below the average for sub-Saharan Africa. Less than 5% of Nigerians have health insurance coverage, as most enrolees are in the formal sector with very poor coverage in the informal sector [24].

### Protocol registration

The study protocol was prospectively registered in the international prospective register of systematic review, PROSPERO (CRD42020161456). The conduct and reporting of this systematic review and meta-analysis have been guided by the Preferred Reporting Items for Systematic Reviews and Meta-Analyses (PRISMA; Additional file 1) [25].

### Search strategy

We conducted a search of previous systematic reviews and protocols related to the topic of interest using PROSPERO database and database of abstracts of review of effects (DARE) (https://www.inahta.org/hta-database/) to confirm a similar study had not been previously registered and published. A comprehensive search of relevant peer-reviewed articles published between January 1, 2010 and December 31, 2019, was conducted in PubMed, Google Scholar and African Index Medicus. We consulted an expert generalist librarian to develop a search strategy using medical subject headings (MeSH) terms, Boolean functions (AND, OR) and keywords related to HBV and prevalence (Additional file 2). Following an initial search string in PubMed, search strategies were adapted for each database. No language limits were applied. Articles were also identified by snow-balling through references of included full-text studies. We used Endnote X9 to catalogue publications and manage references.

### Inclusion and exclusion criteria

Studies that reported the prevalence of HBV in different regions of Nigeria using valid HBV tests were included in this systematic review. Valid HBV tests detect viral proteins (antigens), the antibodies that are produced in response to an infection, or detect the genetic material (DNA) of the virus. The searches were limited by publication year from 2010, in order to specifically focus on studies conducted in the last decade—a decade that succeeded the introduction of HBV vaccine to Nigeria. Further, Musa had earlier conducted a meta-analysis of data published between 2000 and 2013, with 61% of the data included collected prior to 2010 [14]. Systematic reviews or meta-analyses, case studies, surveillance reports, conference abstracts, animal studies, or studies that did not contain HBV prevalence data were not included in the study.

### Study selection and screening

We used Covidence, a software for the management of systematic reviews [26] to screen for articles, remove duplicates and streamline the process of study selection. Two reviewers (BIA and IY) independently screened the titles and abstract, and conducted a full-text review of articles that appeared to meet the inclusion criteria. Disagreements were resolved through mutual consensus, or if required, with consultation with the senior author (BAL).

### Data extraction

A data extraction form was piloted and fields were refined in consultation with several authors (BIA, IY, KR, AR, BAL). Data extraction fields included standard information such as, general study descriptors (year of publication, region, setting, study design), population characteristics (study group, age), and the number of HBV positive cases, the participant sample size, and the method of diagnosis. Data were extracted independently using a Microsoft Excel Sheet (version 16). Author BIA and IY extracted data from all articles. The senior author was consulted to resolve any differences.

### Quality assessment

We used the Joanna Briggs Institute checklist for prevalence studies to critically appraise [27] the overall methodological quality of included studies. The critical appraisal tool included nine parameters which had yes, no, unclear and not applicable options. The parameters evaluated were: appropriateness of the sampling frame, proper sampling technique, adequacy of sample size, study subject and setting description, sufficient data analysis, use of valid methods for the identified conditions, measurement of the condition in a standard and reliable way, the appropriateness of statistical analysis, and the adequacy of the response rate (Additional file 3). A score of 1 was assigned for a ‘yes’ response and 0 was assigned for ‘no’ and ‘unclear’ responses. The mean score was computed for each study. Two reviewers (BIA and IY) independently conducted the quality assessment, and disagreements were resolved by consensus.

### Data analysis

We analysed meta-data using the  metafor package of the statistical software R-version 4.0.2 [28]. Prevalence was defined as the rate of positive assays for HBV. Data were pooled within a random effects model, and a test for heterogeneity was evaluated by the χ^2^ test on Cochrane’s Q statistic [29], quantified by I^2^ value. The I^2^ statistic estimates the percentage of total variation across studies due to true between-study differences rather than chance. The degree of heterogeneity is classified to three levels (minimal, I^2^ < 25%; moderate, 25% ≤ I^2^ < 50%; substantial, and I^2^ ≥ 50%) [30]. When substantial heterogeneity is detected, a random effects model is recommended for estimating HBV pooled prevalence at 95% CI instead of a fixed-effects model [31]. We assessed the sources of variation among studies with sub-group analysis using the following grouping variables: year of publication, study group, age, region, setting, method of diagnosis, and methodological quality. Publication bias was evaluated using funnel plot asymmetry and the Egger’s test [32]. *p*-value < 0.05 on the Egger’s test was considered indicative of statistically significant publication bias. Cohen’s kappa coefficient (k) was calculated to see if there was inter-rater agreement between the two reviewers (BA and IY) in abstract screening and methodological quality assessment.

We used the ESRI package in ArcGIS to map the geographical locations of included studies and the regional prevalence of HBV in Nigeria [33].

## Results

### Study selection

**Fig. 1:**
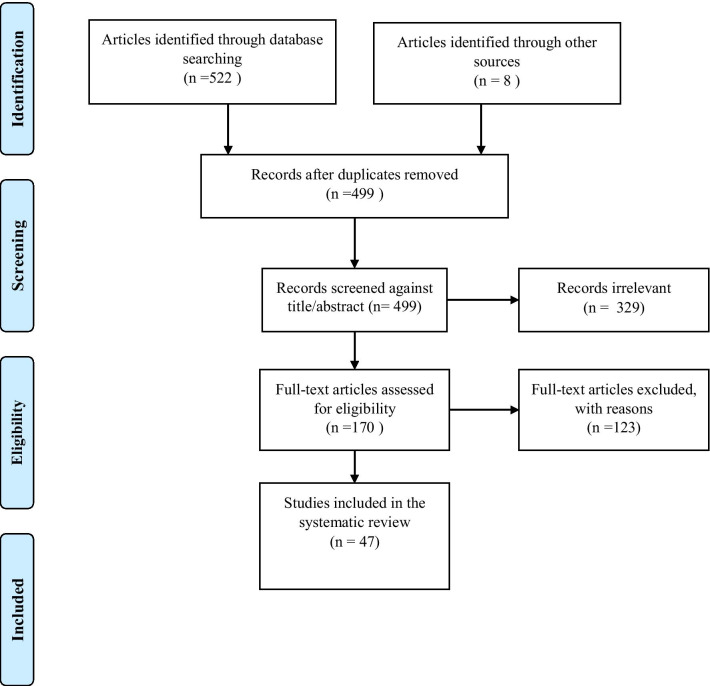
PRISMA flowchart for literature search and study selection

PRISMA flow chart for study selection is presented in Fig. 1. We identified 522 records from the three databases including 248 from PubMed, 33 from African Index Medicus and 241 from Google Scholar. An additional eight studies were identified from screening the bibliography of eligible studies. A total of 47 studies met the inclusion criteria and were included in the systematic review and meta-analysis. A substantial inter-rater agreement between reviewers (BAL, IY) was observed in abstract screening k = 76.8; *p* < 0.01. The *p*-value suggests that the appraiser agreement is significantly different to what would be achieved by chance.

### Characteristics of included studies

A total of 21,702 subjects were covered by the 47 studies, with the sample size in individual studies ranging from 93 to 2391, a median of 300 and mean of 461.7. There was a wide variation of HBV prevalence among included studies, ranging from 1 to 28.4%.

The age range of the enrolled subjects was provided by 87.2% of the studies with the widest age tranche being 1.4–67 years while the narrowest was 5–12 years. Many studies did not report the mean or median age of the sample. But for the purpose of the analyses, we grouped participants into two distinct age categories, namely, children ≤ 17 and adult > 17, based on the premise that HBV vaccination was introduced to Nigeria in 2004. The age distribution of the studies included 6/47 (12.8%) studies in children, 12/47 (25.5%) studies in adult, and 23/47 (48.9%) studies in both children and adult. However, 6/47 studies (12.8%) did not report any age data.

Four (8.5%) studies were conducted amongst blood donors, 7/47 (14.9%) studies amongst HIV positive individuals and 2/47 (4.3%) amongst HIV positive pregnant women. The majority of the studies 17/47 (36.1%) involved the general population, and pregnant women 17/47 (36.1%).

The earliest study was conducted in 2010 and the latest in 2019 (Table 1). The number of articles published increased nearly two-fold from 18 (38.3%) during the 2010–2014 publication period to 29 (61.7%) from 2015 to 2019. Many studies did not report sex-disaggregated data, hence there was no sufficient information available to stratify the gender profile.

**Table 1:** Characteristics of included studies

Author details	Year of publication	Design	Region of study	Study population	Age group	Setting	Sample size (n)	Diagnosis	HBsAg+ (%)	Quality grade
Aba and Aminu [65]	2016	Cross-sectional	North West	Pregnant women	16–50	Urban	800	ELISA	3.9	8
Abulude et al. [66]	2017	Cross-sectional	North West	Pregnant women	NS	Urban	160	ELISA	6.9	6
Adegbesan-Omilabu et al.[67]	2015	Cross-sectional	South West	Pregnant women	18–44	Urban	150	ELISA	7.3	8
Adekanle et al. [68]	2010	Cross-sectional	South West	Blood donors	18–56	Urban and rural	234	ELISA	17.1	6
Adeyemi et al. [69]	2014	Cross-Sectional	South West	Pregnant women	16–35	Urban and rural	658	ELISA	16.3	7
Adoga et al. [70]	2010	Cross-sectional	North Central	Apparently healthy	≤ 60	Urban	1891	ELISA	6.0	6
Alagbeleye et al. [71]	2013	Cross-sectional	South South	Pregnant women	20–45	Urban	250	RDT	6.0	7
Anaedobe et al. [72]	2015	Cross sectional	South West	Pregnant women	22–44	Urban	180	PCR	8.3	7
Anigilaje et al.[73]	2013	Cross sectional	North Central	HIV positive	8 months-15 years	Urban	395	ELISA	7.8	7
Atilola et al. [74]	2018	Cross-sectional	South West	Pregnant women	15–49	Urban	353	RDT	10.5	6
Augustine et al. [75]	2014	Cross-sectional	North West	Blood donors	18–65	Urban	150	RDT	9.3	6
Babatope et al. [76]	2015	Cross-sectional	South South	Apparently healthy	NS	Urban	300	RDT	8.3	6
Balogun et al. [77]	2010	Cross-sectional	South West	HIV positive	> 18	Urban	102	ELISA	28.4	6
Bakarey et al. [78]	2018	Cross-sectional	South West	Chronic liver disease	20–60	Urban	122	ELISA	13.9	7
Esan et al. [79]	2014	Cross-sectional	South West	Pregnant women	> 15	Urban	649	ELISA	6.8	6
Ezechi et al. [80]	2014	Cross-sectional	South West	HIV positive pregnant women	14–44	Urban	2391	ELISA	4.20	7
Frank-Peterside et al. [81]	2016	Cross-sectional	South South	HIV positive	> 20	Urban	535	RDT	4.67	5
Godwin et al. [82]	2017	Cross sectional	North Central	Students	9–24	Urban	158	RDT	13.3	5
Habibu et al. [83]	2017	Cross-sectional	North West	Students	5–12	Rural	240	RDT	23.3	7
Ifeorah et al. [84]	2017	Cohort	North Central	HIV positive	1.4–67	Urban	1102	ELISA	10.3	7
Iklaki et al. [85]	2015	Cross-sectional	South South	Pregnant women	16–43	Urban and rural	300	ELISA	4.7	7
Kolawole et al. [86]	2012	Cross-sectional	South West	Pregnant women	> 15	Urban and rural	200	ELISA	16.5	8
Kolawole et al. [87]	2018	Cross-sectional	North Central	Febrile patients	> 17	Urban	200	EIA	19.0	6
Meka et al. [88]	2019	Cross-Sectional	South East	Apparently healthy	NS	Rural	330	RDT	2.1	6
Mohammed et al. [89]	2019	Cross-sectional	North Central	Students	> 15	Urban	350	RDT	9.7	6
Motayo et al. [90]	2015	Cross-sectional	South West	Blood donors	NS	Urban	305	EIA	9.8	7
Ndako et al. [91]	2010	Cross-sectional	North Central	Students	4–17	Rural	360	RDT	9.7	7
Ndako et al. [92]	2011	Cross-sectional	North West	Students	10–24	Rural	190	RDT	18.4	5
Ndako et al. [93]	2016	Cross-sectional	North Central	Apparently healthy	> 25 years	Urban and rural	200	ELISA	22.5	6
Nejo et al. [94]	2018	Cross-sectional	South West & North Central	Sexually active	15–60	Urban and rural	463	ELISA	10.4	7
Ngwogu et al. [95]	2016	Cross-sectional	South East	HIV positive	NS	Urban	306	RDT	9.8	4
Oje et al. [96]	2012	Cross-sectional	South West	Apparently healthy	≥ 15	Urban and rural	2000	RDT	9.8	6
Okonko et al. [97]	2011	Cross-sectional	South West	Pregnant women	NS	Urban	200	RDT	11.5	7
Okoye et al. [98]	2015	Cross-sectional	North Central	HIV positive pregnant women	21–50	Urban	124	ELISA	12.1	6
Olayinka et al. [99]	2016	Cross-sectional	Country-wide	Apparently healthy	> 2	Urban and rural	965	ELISA	12.2	8
Oluboye et al. [100]	2014	Cross-sectional	South East	Pregnant women	≥ 15	Urban	100	ELISA	6.0	4
Omatola et al. [101]	2019	Cross-sectional	South West	Pregnant women	≥ 15	Urban	200	RDT	1.0	7
Omeje et al. [102]	2017	Cross-sectional	South East	Students	9–23	Urban	266	RDT	3.4	8
Onwuakor et al. [103]	2014	Cross-sectional	South East	Pregnant women	17–46	Urban	350	RDT	7.1	6
Onwuliri et al. [104]	2017	Cross-sectional	North Central	Pregnant women	15–54	Urban and rural	406	EIA	10.0	4
Osho et al. [105]	2019	Cross-sectional	South West	Pregnant women	≥ 17	Urban	1758	RDT	4.2	4
Oyinloye et al. [106]	2018	Cross-sectional	North East	Outpatient	15–64	Urban and rural	410	RDT	7.1	5
Rabiu et al. [107]	2018	Cross-sectional	North West	Pregnant women	18–54	Urban and rural	93	ICT	17.2	6
Sadoh et al. [108]	2011	Cross-sectional	South South	HIV positive	2 months–17 years	Urban	155	ELISA	7.7	7
Sadoh et al. [109]	2014	Cross-sectional	South South	Children admitted to emergency unit	2 months–15 years	Urban	150	ELISA	14.0	7
Uleanya [110]	2016	Cross-sectional	South East	HIV positive	18 months–15 years	Urban	140	EIA	10.0	6
Yakubu et al. [111]	2016	Cross-sectional	North West	Blood donors	18–35	Urban and rural	361	RDT	16.6	5

*HBsAg* Hepatitis B surface antigen, *NS* not specified, *NA* Not available, *ELISA* enzyme-linked immunosorbent assay, *PCR* polymerase chain reaction*, ICT* immunochromatography, *EIA* enzyme immunoassay, *RDT* rapid diagnostic test

**Fig. 2:**
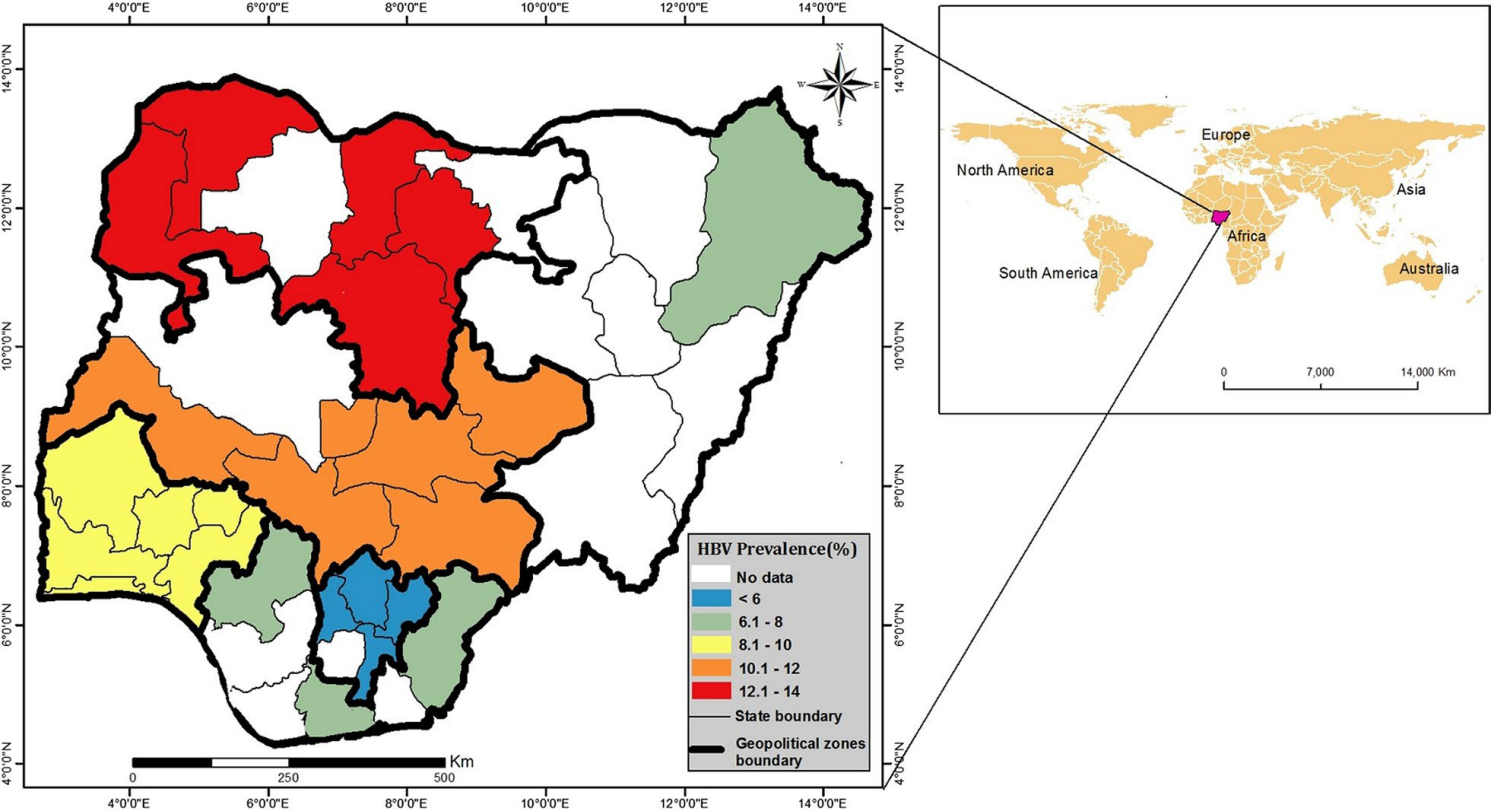
Map of Nigeria showing the geographical locations of included studies and the regional prevalence of HBV

The included studies captured the six geo-political zones of Nigeria. Of these, two studies had a nationwide and inter-regional coverage. The regional locations of the studies included 10/47 (21.3%) studies in the North Central zone, 1/47 (2.1%) study in the North East zone, 7/47 (14.9%) studies in the North West zone, 6/47 (12.8%) studies in the South East zone, 6/47 (12.8%) studies in the South South, 15/47 (31.9%) studies in the South West zone, and 2/47 (4.3%) studies with a mixed coverage. Another 4/47 (8.5%) studies were conducted in rural populations, 31/47 (66.0%) in urban populations and 12/47 (25.5%) in mixed populations. The regional prevalence of HBV is shown in Fig. 2.

Examining the methods of diagnoses used for detecting HBV, 21/47 (44.7%) studies used enzyme-linked immunosorbent assay (ELISA). Rapid diagnostic test (RDT) was used in 20/47 (42.5%) studies, while polymerase chain reaction (PCR) was used in only one study (2.1%).

### Methodological quality of included studies

The studies scored median of 6 (range 4–8) using JBI’s nine items of risk bias (Additional file 4). Five (10.6%) studies had a quality score of 8, 38/47 (80.9%) studies scored between 5 and 7, and 4/47 (8.5%) studies had a quality score of 4. All studies used valid diagnostic methods for HBV detection and the prevalence was measured in a standardised way. An almost perfect inter-rater agreement of k = 84.9%; p < 0.01 was observed between the two reviewers (BA and IY).

### Prevalence of HBV infection in Nigeria

**Fig. 3:**
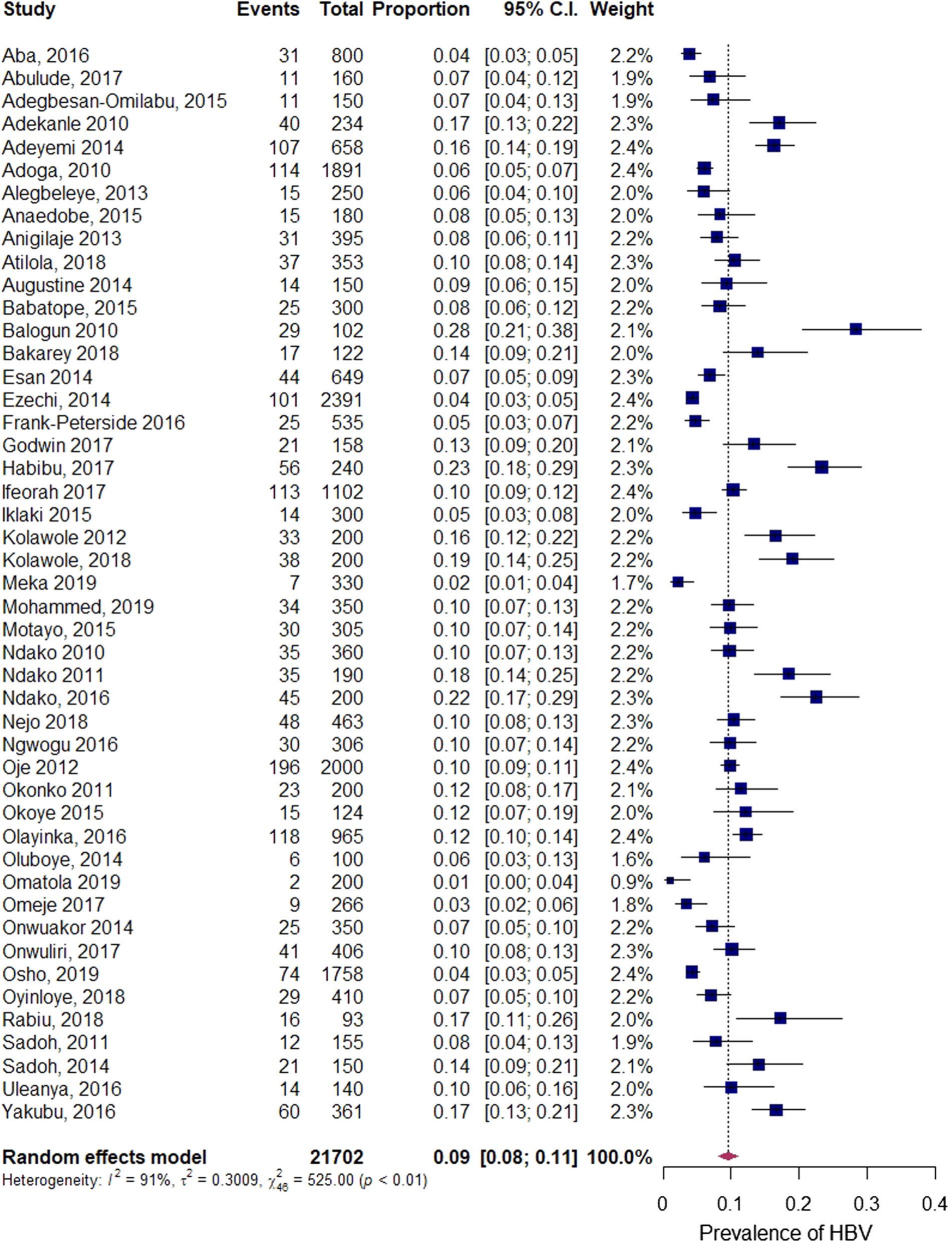
Forest plot of the pooled prevalence of HBV in Nigeria from 2010 to 2019

The pooled prevalence of HBV infection was 9.5% (95% CI 8.1–11.0), with heterogeneity index (*I*^2^) of 91.2% (*p* < *0.01*) (Fig. 3), confirming substantial heterogeneity across studies.

### Subgroup analysis of HBV prevalence

The results of the sub-group analysis of the pooled prevalence, including assessment of differences between sub-groups, are presented in Table 2. Geographically, the North West Zone had the highest prevalence (12.1%, 95% CI 7.2–19.6) while the South East Zone had the lowest prevalence (5.9%, 95% CI 3.8–9.2). In the studies providing age-specific estimates, the prevalence was higher in adults (12.7%, 95% CI 8.4–18.8) than in children (11.4%, 95% CI 7.5–17.1). Studies conducted on blood donors accounted for the highest prevalence (13.2%, 95% CI 9.7–17.8) followed by other general population (10.8%, 95% CI 8.6–13.5) and HIV positive individuals (9.9%, 95% CI 6.7–14.4). Subgroup analysis by publication year revealed an apparent downward trend in prevalence from 2010 to 2014 (10.1%, 95% CI 7.9–13.0) to 2015–2019 group (9.0%, 95% CI 7.4–11.0). Further, studies conducted in urban areas showed lower prevalence (8.2%, 95% CI 6.9–9.9) than studies conducted in rural areas (10.7%, 95% CI 5.01–21.4). In reference to the methods of diagnosis, studies conducted using PCR accounted for the least prevalence estimate (8.3%, 95% CI 5.1–13.4). A relatively large degree of heterogeneity was identified across most estimates. Setting (*p* = 0.008) and region (*p* = 0.005) were significantly associated with the pooled prevalence heterogeneity (*p* < 0.05).

**Table 2:** Sub-group analysis of the pooled prevalence of hepatitis B infection in Nigeria

Sub-group variables	Variable category	Included studies	Prevalence % (95% CI)	I^2^%	*p*-heterogeneity	*p-*difference
Study group	Blood donor	4	13.2 (9.7–17.8)	72.5	0.012	0.129
	Pregnant women	17	7.7 (5.8–10.2)	90.1	*p* < 0.001	
	HIV positive	7	9.9 (6.7–14.4)	88.6	*p* < 0.001	
	HIV positive pregnant women	2	7.0 (2.4–18.7)	93.3	*p* < 0.001	
	Others	17	10.8 (8.6–13.5)	91.1	*p* < 0.001	
Mean methodological quality	JBI ≤ 6	9	9.1 (6.0–13.4)	92.7	*p* < 0.001	0.795
	JBI > 6	38	9.6 (8.1–11.3)	91.0	*p* < 0.001	
Region/ zone	North West	7	12.1 (7.2–19.6)	93.3	*p* < 0.001	0.005
	North East	1	7.1 (5.0–10.0)	NA	1.0	
	South West	15	9.7 (7.1–13.1)	94.3	*p* < 0.001	
	South East	6	5.9 (3.8–9.2)	76.7	*p* < 0.001	
	South South	6	7.1 (4.9–10.0)	73.6	0.002	
	North Central	10	11.2 (8.5–14.7)	89.6	*p* < 0.001	
	Mixed	2	11.6 (10.0–13.5)	5.0	0.305	
Year of publication	2010–2014	18	10.1 (7.9–13.0)	92.6	*p* < 0.001	0.467
	2015–2019	29	9.0 (7.4–11.0)	90.4	*p* < 0.001	
Method of diagnosis	ELISA	21	10.0 (7.8–12.6)	93.2	*p* < 0.001	0.066
	RDT	20	8.4 (6.5–10.6)	90.7	*p* < 0.001	
	PCR	1	8.3 (5.1–13.4)	NA	NA	
	EIA	4	11.9 (8.4–16.7)	75.6	0.006	
	ICT	1	17.2 (10.81–26.3)	NA	NA	
Setting	Rural	4	10.7 (5.01–21.4)	94.3	*p* < 0.001	0.008
	Urban	31	8.2 (6.9– 9.9)	88.0	*p* < 0.001	
	Mixed	12	12.6 (10.3–15.3)	86.9	*p* < 0.001	
Age	≤ 17	6	11.4 (7.5–17.1)	87.2	*p* < 0.001	0.140
	> 17	12	12.7 (8.4–18.8)	94.2	*p* < 0.001	
	Mixed	23	8.2 (6.8–10.0)	90.8	*p* < 0.001	
	Not stated	6	7.8 (5.5–10.9)	73.2	*0.002*	

*ELISA *enzyme-linked immunosorbent assay, *PCR* polymerase chain reaction, *ICT *immunochromatography,* EIA *enzyme immunoassay, *RDT* rapid diagnostic test

### Publication bias

**Fig. 4:**
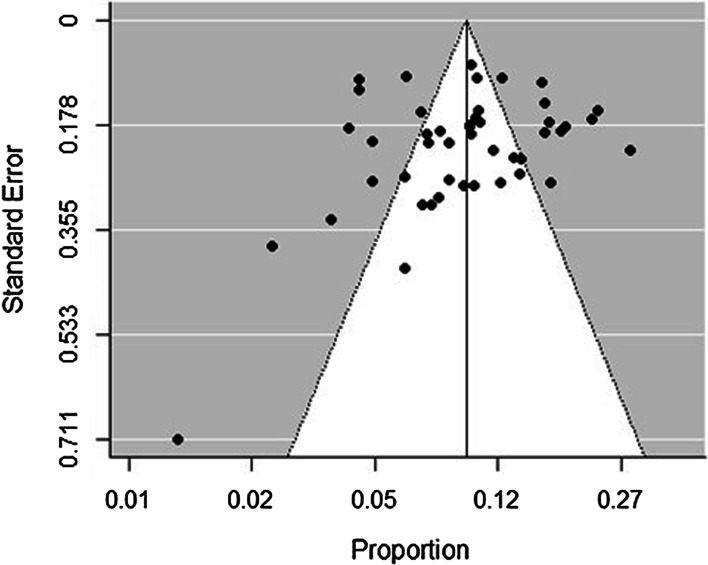
Bias assessment funnel plot of studies reporting HBV prevalence in Nigeria from 2010 to 2019

The Egger’s regression test was not statistically significant (p = 0.879). We found no evidence of publication bias, as supported by the funnel plot (Fig. 4).

## Discussion

In this meta-analysis, we set out to determine the pooled prevalence of HBV in the Nigerian population. Our analysis shows that HBV prevalence is high, as per the World Health Organisation’s criteria for HBV endemicity, (≥ 8%; high, 2–7%; moderate and < 2%; low) [34]. Findings of this study provide a critical data to assess the impact of current prevention and control strategies in Nigeria, and serve as a reference for designing and implementing effective public health management programmes towards the 2030 elimination goal.

Nigeria’s overall pooled prevalence of HBV 9.5% is similar to that of Cameroon 11.2% [35] and Burkina Faso 11.2% [36] but greater than that of Ethiopia 6% [37]. The United Nations estimates that Nigeria’s population in 2021 is 211.4 million [38]. Applying the 9.5% prevalence to this figure translates into 20.083 million people who had HBV. Somehow, these millions have been missing in the public health space, not only in Nigeria but also in the global public health agenda [39]. Until recently when the WHO set the 2030 elimination goal for viral hepatitis, HIV/AIDS which affects an estimated 1.9 million persons in Nigeria [40] had been receiving more public health priority. In Nigeria, the burden of viral hepatitis B requires continued improvement of screening, broader access to treatment and the integration of prevention and care in local healthcare systems. The WHO’s ambitious goal to eliminate viral hepatitis by 2030 may not come into fruition if the trends in healthcare system are not realigned towards universal care in the face of this high prevalence.

The pooled prevalence estimates of HBV among pregnant women accounted for 7.7%. In Burkina Faso, a prevalence of 11.2% [36] was obtained among pregnant women. Jean et al. [35] and Ofori-Asenso et al. [41] also obtained a prevalence of 11.1% and 13.1% among pregnant women in Cameroon and Ghana, respectively. Though, the obtained prevalence for pregnant women in this study is lower than the aforementioned countries, it is higher than the prevalence in Ethiopia 5.0% [42], Rwanda 3.1% [43] and Tanzania 5.2% [44]. The prevalence in the sub-population of pregnant women in Nigeria has serious implications, including the risk of perinatal transmission of HBV infection and accelerated HBV-related liver disease. Robust pre-conception screening and the implementation of ‘test and treat’ interventions at low cost for infected couples can bolster the existing policy on the prevention of vertical transmission of HBV. In addition, timely birth dose vaccination of newborn infants is critical to provide the earliest possible protection to future birth cohorts and reduce the pool of chronic HBV carriers in the population.

HBV prevalence among patients with HIV was estimated to be 9.9%, although this rate is lower than the 12.5% reported in a study by Hamza et al. [45], and the 11.5% prevalence obtained from a health facility in North central Nigeria [46]. This prevalence is higher than the WHO’s threshold for high-endemic areas. Additionally, this study revealed a prevalence of 7.0% among HIV positive pregnant women. These results suggest that the burden of HBV and HIV co-infection is significant in the Nigerian population, and may lead to higher levels of detectable virus [47], accelerated cirrhosis [48] and increased likelihood of developing HCC [49] in the already immune-compromised sub-population. The dynamics and epidemiology of HBV infection is distinct in HIV patients, and continuing to target these individuals with the implementation of HBV screening remains very pertinent.

HBV prevalence among blood donors was estimated to be 13.2%, although this estimate is lower than the 14.0% reported in a previous systematic review by Musa et al. [19]. This result raises significant concern regarding the safety of blood and blood products in Nigeria, as nearly 1 in 8 blood donors may be infected with HBV. Our results further showed that ELISA and RDTs were the most frequently used diagnostics in the Nigerian population, including blood donors. This could be attributed to their relative ease of use and lower cost. Meanwhile, the less than 60% sensitivity reported for RDTs, and the inability of ELISA to detect certain HBV mutant strains have raised serious concerns for their use in blood donation centres [50]. This emphasises the need for robust screening techniques and strict adherence to the national blood transfusion policy in Nigeria, which requires the screening of all donated blood for transfusion-transmissible infections including HIV, HBV, HCV and syphilis [51].

As revealed by this study, we found region-specific differences in prevalence estimates. The highest 12.1% and the least prevalence 5.9% were obtained from the North West and South East geo-political zones, respectively. This is consistent with patterns previously described in literature, with the potential for marked differences in prevalence, even between neighbouring states [52]. A diverse range of factors influence the observed variations in HBV prevalence across Nigeria, with significant inequities in birth dose vaccination, among the most widely recognised. The immunisation coverage of birth dose varied widely across Nigeria, from the highest coverage of 64.9% in South East to the lowest coverage of 14.1% in North West [53]. Multiple barriers have been identified for the low birth dose uptake in North West region [54], including relative geographical isolation, religious beliefs that fuel vaccine hesitancy, limited antenatal screening for HBV surface antigen, birth occurring outside healthcare facilities, and lack of skilled medical staff to provide birth-dose vaccination.

Of particular interest from our findings is the rural–urban difference in the prevalence of HBV. We obtained a prevalence of 10.7% in rural setting, compared with 8.2% in urban setting. This indicates that HBV is most prevalent in rural areas of Nigeria, and may have been intensified by the lack of awareness about the implications of HBV infection, under-developed shared care pathways for HBV management, and high-risk lifestyles that characterise the countryside. This result suggests the need to develop trust and ensure culturally appropriate strategies when delivering care for HBV in rural settings.

When stratified by age, the prevalence estimate was 12.7% in the age group > 17, namely, those born before 2004, then decreased to 11.4% in the age group ≤ 17. While this was not significant noting the overlapping confidence intervals and *p* of 0.140, it is worth noting that these are individuals born from 2004 upwards, the year which marked the implementation of the universal HBV immunisation programme in Nigeria. The observed decline in prevalence may reflect the increase in HBV vaccination coverage among children. UNICEF reported HBV vaccination coverage was 0% in 2000–2005, 18% in 2006 [55], and 41% in 2013 [19]. However, there was a significant drop in vaccination coverage from 41% in 2013 to 30.2% in 2016/2017 [53]. Despite the reduced coverage level, the importance of infant vaccination remains very paramount, as validated in clinical practice and population epidemiology across diverse settings.

Further, the observed prevalence in the children age-group presents an evidence of a continued acquisition of HBV infection in the vaccine-eligible population. Beyond low vaccination coverage, the most likely explanation is an incomplete vaccine coverage [56, 57]. Research has shown that infants who received fewer than the three scheduled hepatitis B vaccine doses are at a higher risk of infection [58, 59]. Inadequate coverage of the full schedule may have contributed to the rate of infection observed in vaccine-eligible child populations in Nigeria. This emphasises the need for Nigeria to take pragmatic measures in meeting the WHO target of 90% coverage of the hepatitis B vaccine in infancy [60], as low and inadequate coverage are potential contributors to high HBV prevalence.

Unlike hepatitis C, there is no therapeutic for hepatitis B, but current treatments are well tolerated and effective at preventing liver disease and reducing viral load. It is believed that 9 in 10 Nigerians living with HBV are unaware of their infection status, and therefore at risk of not receiving treatment due to inadequate monitoring for their condition [61]. This profound lack of access to treatment and care among the adult population, with a prevalence of 12.7% contributes to the disproportionate impact of HBV in Nigeria.

In this study, HBV prevalence appears to be declining over time. This is consistent with global trends [39]. Studies conducted between 2000 and 2013 by Musa et al. [19] recorded the highest prevalence of 14.0%, compared with the lower prevalence estimates of 10.1% and 9.0% that we obtained for studies between 2010–2014 and 2015–2019, respectively. These apparent downward trend in prevalence of HBV in Nigeria may in part reflect coverage of the universal HBV vaccination introduced in 2004, and the likely improvements in socio-economic conditions across time-period. The results suggest that to further reduce HBV prevalence in Nigeria, a dedicated focus on socio-economic, cultural and population health factors are required, for example, equity of access across rural and urban regions.

Among methods of diagnosis, PCR accounted for the lowest prevalence estimate of 8.3%, compared with ELISA 10.0%. This result further suggests that PCR was the least used method of diagnosis in the Nigerian population. This reflects the high-cost implication of PCR. There are relatively few laboratories in Nigeria that possess the capability of performing real-time quantitative PCR for HBV DNA. The cost of this assay is about $200–$250 per test, and as in many settings, patients have to pay for the test. It also requires time, skilled lab technicians, and sophisticated equipment and laboratories, and the value is used in the algorithm for making decision on who to treat and how to clinically manage the patients, in line with the international criteria for treatment eligibility [62]. Therefore, most infected populations are left without care, since treatment eligibility rely on parameters such as, HBV viral load and liver fibrosis, and these are often difficult to assess routinely in resource-constrained settings.

Also, most rural and remote laboratories do not have access to specialised second-tier assays, such as immunoassays. These limitations have impeded the uptake of HBV screening in the Nigerian population. Consequently, there is an urgent need for alternative diagnostics that assess and/or predict HBV infection, in a way that will improve access to affordable testing for the missing millions who are unaware of their HBV status. For instance, a simple pathology index, the gamma-glutamyl transpeptidase to platelet ratio (GPR) [63] was developed in HBV-monoinfected subjects in The Gambia, with improved performance for the diagnosis of fibrosis and cirrhosis in comparison to liver biopsy, and with the added advantage for remote and rural settings of not requiring invasive biopsy procedures.

### Strength and limitations

This review and meta-analysis presents the most updated pooled estimate of the prevalence of HBV in Nigeria. Further, it employed a comprehensive search strategy across three key data sources, and involved a large number of studies and study participants, covering the country’s six geopolitical zones. Our estimates were robust and corroborated by the absence of evidence of a publication bias. We also provided the prevalence among age-specific group born before and after the introduction of universal HBV vaccination, to reaffirm the importance of childhood immunisation. This was not investigated by the previous systematic review [19].

Limitations of this systematic review are related to the available data and the substantial heterogeneity across included studies. First, the quality of reporting of studies varied. For instance, many available studies did not report sex-specific prevalence. Further, data available on prevalence in infants were particularly sparse, hence, we could not stratify the available age-banded data to estimate prevalence in under 5 years of age. Second, there was substantial heterogeneity across the included studies, and this may undermine confidence in the pooled estimate. However, we investigated the potential sources of heterogeneity, and results showed that heterogeneity may have been due to the differences in the study setting and region. High levels of heterogeneity appear to be the bane of similar meta-analytic studies on HBV in Africa with reports of I^2^ statistics of 94–99.9% [36, 37, 64].

## Conclusions

Our results show that the burden of HBV infection in Nigeria is high, and is unevenly distributed among various sub-populations and geo-political zones. Further studies are required to better understand the extent to which genotype-specific epidemiological factors might influence the regional distribution of HBV prevalence in Nigeria. To promote future research, and subsequent high-quality translation to health settings, we suggest that national guidelines be developed to ensure consistency for data collection across the country, and hence improve the quality of reporting. For example, the routine recording of age and sex, among other relevant population and individual variables, will support deeper insights into the true HBV situation, and lead to tangible improvements for patients and their communities. We also advocate significant investment in capacity building for improving HBV diagnosis, and sustained surveillance to monitor progress towards elimination. A systematic commitment to early diagnosis and clinical management represents a key component of the journey towards HBV elimination by 2030.

## Supplementary Information

**Additional file 1.** PRISMA 2009 checklist.

**Additional file 2. **Pubmed search strategy.

**Additional file 3.** JBI critical appraisal checklist for studies reporting prevalence data.

**Additional file 4.** Quality assessment of included studies.

## Data Availability

All data generated or analysed during this study are included in this published article (Table 1).

## Abbreviations

HBV: Hepatitis B virus
HCC: Hepatocellular carcinoma
ELISA: Enzyme-linked immunoassay
RDT: Rapid diagnostic test
PCR: Polymerase chain reaction
CI: Confidence interval
