# Supplementary material for: Hepatitis B virus infection in Nigeria: a systematic review and meta-analysis of data published between 2010 and 2019
Source: BMC Infect Dis. 2021 Oct 30;21:1120. doi: 10.1186/s12879-021-06800-6 (PMC8556927; doi:10.1186/s12879-021-06800-6)
Supplement: Supplementary file 2 — Additional file 2. Pubmed search strategy. [file 12879_2021_6800_MOESM2_ESM.doc]

**Additional file 2: Pubmed search strategy**

*(((((((((((("hepatitis b surface antigens"[MeSH Terms] OR "hepatitis b"[MeSH Terms]) OR "hepatitis b virus"[MeSH Terms]) AND "prevalence"[MeSH Terms]) OR "seroepidemiologic studies"[MeSH Terms]) OR "epidemiology"[MeSH Terms]) AND LFTs[MeSH Terms])) OR alanine transaminase[MeSH Terms]) OR alkaline phosphatase[MeSH Terms]) OR liver biomarkers[MeSH Terms]) OR biomarkers, liver diagnosis[MeSH Terms] AND "Nigeria"[MeSH Terms] AND ("2010/01/01"[PDAT] : "2019/12/31"[PDAT])))*
