# Supplementary material for: Hepatitis B virus infection in Nigeria: a systematic review and meta-analysis of data published between 2010 and 2019
Source: BMC Infect Dis. 2021 Oct 30;21:1120. doi: 10.1186/s12879-021-06800-6 (PMC8556927; doi:10.1186/s12879-021-06800-6)
Supplement: Supplementary file 3 — Additional file 3. JBI critical appraisal checklist for studies reporting prevalence data. [file 12879_2021_6800_MOESM3_ESM.doc]

**Additional file 3**

**JBI Critical Appraisal Checklist for Studies Reporting Prevalence Data**

Reviewer Date

Author Year Record Number

|  | Yes | No | Unclear | Not applicable |
| --- | --- | --- | --- | --- |
| 1. Was the sample frame appropriate to address the target population? | □ | □ | □ | □ |
| 1. Were study participants sampled in an appropriate way? | □ | □ | □ | □ |
| 1. Was the sample size adequate? | □ | □ | □ | □ |
| 1. Were the study subjects and the setting described in detail? | □ | □ | □ | □ |
| 1. Was the data analysis conducted with sufficient coverage of the identified sample? | □ | □ | □ | □ |
| 1. Were valid methods used for the identification of the condition? | □ | □ | □ | □ |
| 1. Was the condition measured in a standard, reliable way for all participants? | □ | □ | □ | □ |
| 1. Was there appropriate statistical analysis? | □ | □ | □ | □ |
| 1. Was the response rate adequate, and if not, was the low response rate managed appropriately? | □ | □ | □ | □ |

Overall appraisal: Include □ Exclude □ Seek further info □

Comments (Including reason for exclusion)
