# Supplementary material for: Hepatitis B virus infection in Nigeria: a systematic review and meta-analysis of data published between 2010 and 2019
Source: BMC Infect Dis. 2021 Oct 30;21:1120. doi: 10.1186/s12879-021-06800-6 (PMC8556927; doi:10.1186/s12879-021-06800-6)
Supplement: Supplementary file 4 — Additional file 4. Quality assessment of included studies. [file 12879_2021_6800_MOESM4_ESM.doc]

Additional file 4: Quality assessment of included studies.

| **Study** | **Q1** | **Q2** | **Q3** | **Q4** | **Q5** | **Q6** | **Q7** | **Q8** | **Q9** | **Score** |
| --- | --- | --- | --- | --- | --- | --- | --- | --- | --- | --- |
| Aba, 2016 | Y | UC | Y | Y | Y | Y | Y | Y | Y | 8 |
| Abulude, 2017 | N | Y | N | Y | Y | Y | Y | Y | UC | 6 |
| Adegbesan-Omilabu, 2015 | Y | UC | Y | Y | Y | Y | Y | Y | Y | 8 |
| Adekanle, 2010 | N | UC | N | Y | Y | Y | Y | Y | Y | 6 |
| Adeyemi, 2014 | UC | Y | Y | Y | Y | Y | Y | Y | UC | 7 |
| Adoga, 2010 | N | UC | N | Y | Y | Y | Y | Y | Y | 6 |
| Alagbeleye, 2013 | N | UC | Y | Y | Y | Y | Y | Y | Y | 7 |
| Anaedobe, 2015 | Y | N | N | Y | Y | Y | Y | Y | Y | 7 |
| Anigilaje, 2013 | Y | Y | Y | Y | N | Y | Y | Y | N | 7 |
| Atilola, 2018 | Y | Y | N | Y | Y | UC | UC | Y | Y | 6 |
| Augustine, 2014 | N | UC | N | Y | Y | Y | Y | Y | Y | 6 |
| Babatope, 2015 | N | UC | N | Y | Y | Y | Y | Y | Y | 6 |
| Balogun, 2010 | N | UC | N | Y | Y | Y | Y | Y | Y | 6 |
| Bakarey, 2018 | Y | UC | N | Y | Y | Y | Y | Y | Y | 7 |
| Esan, 2014 | N | UC | N | Y | Y | Y | Y | Y | Y | 6 |
| Ezechi, 2014 | Y | Y | Y | Y | Y | UC | UC | Y | Y | 7 |
| Frank-Peterside, 2016 | N | UC | N | Y | Y | Y | Y | Y | UC | 5 |
| Godwin, 2017 | N | UC | Y | N | Y | Y | Y | Y | UC | 5 |
| Habibu, 2017 | N | Y | Y | Y | Y | Y | Y | Y | UC | 7 |
| Ifeorah, 2017 | N | Y | UC | Y | Y | Y | Y | Y | Y | 7 |
| Iklaki, 2015 | Y | UC | N | Y | Y | Y | Y | Y | Y | 7 |
| Kolawole, 2012 | Y | UC | Y | Y | Y | Y | Y | Y | Y | 8 |
| Kolawole, 2018 | N | UC | N | Y | Y | Y | Y | Y | Y | 6 |
| Meka, 2019 | N | UC | N | Y | Y | Y | Y | Y | Y | 6 |
| Mohammed, 2019 | N | UC | N | Y | Y | Y | Y | Y | Y | 6 |
| Motayo, 2015 | Y | UC | N | Y | Y | Y | Y | Y | Y | 7 |
| Ndako, 2010 | N | Y | N | Y | Y | Y | Y | Y | Y | 7 |
| Ndako, 2011 | N | UC | N | Y | Y | Y | Y | Y | N | 5 |
| Ndako. 2016 | Y | UC | N | Y | Y | Y | Y | Y | UC | 6 |
| Nejo, 2018 | Y | Y | N | Y | Y | Y | Y | Y | UC | 7 |
| Ngwogu, 2016 | N | UC | N | N | Y | Y | Y | Y | UC | 4 |
| Oje, 2012 | Y | UC | N | Y | Y | Y | Y | Y | UC | 6 |
| Okonko, 2011 | Y | UC | N | Y | Y | Y | Y | Y | Y | 7 |
| Okoye, 2015 | Y | UC | N | Y | Y | Y | Y | Y | N | 6 |
| Olayinka, 2016 | Y | Y | N | Y | Y | Y | Y | Y | Y | 8 |
| Oluboye, 2014 | N | UC | N | N | Y | Y | Y | Y | UC | 4 |
| Omatola, 2019 | N | Y | Y | Y | Y | Y | Y | Y | UC | 7 |
| Omoje, 2017 | Y | Y | Y | Y | Y | Y | Y | Y | N | 8 |
| Onwuakor, 2014 | N | N | N | Y | Y | Y | Y | Y | Y | 6 |
| Onwuliri, 2016 | N | UC | N | N | Y | Y | Y | Y | UC | 4 |
| Osho, 2019 | N | UC | N | N | Y | Y | Y | Y | UC | 4 |
| Oyinloye, 2018 | Y | UC | Y | N | Y | Y | Y | N | UC | 5 |
| Rabiu, 2018 | Y | UC | N | Y | Y | Y | Y | Y | UC | 6 |
| Sadoh, 2011 | Y | UC | Y | Y | Y | Y | Y | Y | UC | 7 |
| Sadoh, 2014 | Y | UC | Y | Y | Y | Y | Y | Y | UC | 7 |
| Uleanya, 2016 | N | UC | N | Y | Y | Y | Y | Y | Y | 6 |
| Yakubu, 2016 | N | UC | N | Y | Y | Y | Y | Y | UC | 5 |

Y: Yes; N: No; UC: Unclear.
